# Tuning Hydrophobicity of Paper Substrates for Effective Colorimetric detection of Glucose and Nucleic acids

**DOI:** 10.1007/s00216-023-04921-2

**Published:** 2023-09-04

**Authors:** Sujesh Sudarsan, Prashil Shetty, Raja Chinnappan, Naresh Kumar Mani

**Affiliations:** 1 Microfluidics, Sensors and Diagnostics (μSenD) Laboratory, Centre for Microfluidics, Biomarkers, Photoceutics and Sensors (μBioPS), Department of Biotechnology, Manipal Institute of Technology, Manipal Academy of Higher Education, Manipal, Karnataka 576104 India; 2 College of Medicine, Alfaisal University, 11533 Riyadh, Saudi Arabia

**Keywords:** Paper-based analytical device, LAMP amplicon, Serum glucose, Hydrophobicity, Colorimetric enhancement

## Abstract

This study investigated the colorimetric response of standard glucose, serum glucose, and nucleic acid assays on various paper surfaces with different wettability, including hydrophilic, hydrophobic, and nearly superhydrophobic surfaces. Water contact angles (WCA) formed by water droplets on each surface were measured using ImageJ software. The hydrophilic surface showed no contact angle, while the hydrophobic and nearly superhydrophobic surfaces exhibited contact angles of 115.667° and 133.933°, respectively. The colorimetric sensitivity of the standard glucose assay was analyzed on these surfaces, revealing enhanced sensitivity on the nearly superhydrophobic surface due to the high molecular crowding effect owing to its non-wetting behavior and eventually confined reaction product at the sample loading zone. The hydrophobic nature of the surface restricts the spreading and diffusion of the reaction product, leading to a controlled and localized concentration of the assay product leading to moderate colorimetric intensity. On the other hand, the hydrophilic surface showed the least enhancement in colorimetric sensitivity; this is attributed to the high wettability of the hydrophilic surface causing the reaction product to spread extensively, resulting in a larger area of dispersion and consequently a lower colorimetric intensity. The measured limit of detection (LOD) for nucleic acid on nearly superhydrophobic surfaces was found to be 16.15 ng/µL, which was almost four-fold lower than on hydrophilic surfaces (60.08 ng/µL). Additionally, the LODs of standard glucose and clinical serum samples were two-fold lower on nearly superhydrophobic surfaces compared to hydrophilic surfaces. Our findings clearly highlight the promising potential of utilizing superhydrophobic surfaces to significantly enhance colorimetric sensitivity in paper-based diagnostic applications. This innovative approach holds promise for advancing point-of-care diagnostics and improving disease detection in resource-limited settings.

**Supplementary Information:**

The online version contains supplementary material available at 10.1007/s00216-023-04921-2.

## Introduction

In the pursuit of improving diagnostic capabilities, researchers have been harnessing the power of biomarkers; the measurable indicators of biological processes or disease states, to revolutionize disease detection methods [1–7]. The past century has seen a rise in various diagnostic strategies for measuring disease-specific signatures in patient samples (e.g., blood, urine, and tissue) [8, 9]. These indicators are typically classified into different types, including genetic, proteomic, and metabolomic biomarkers [10]. In clinical practice, conventional methods such as immunoassays, polymerase chain reaction (PCR), mass spectrometry, and imaging modalities have been the cornerstone of biomarker detection [11, 12]. However, they require complex laboratory procedures and specialized equipment thereby hindering rapid on-site testing [13, 14]. Recently, paper-based point-of-care (POC) diagnostics integrating colorimetric detection modalities have revolutionized biomarker detection by offering unique advantages that address the aforementioned limitations [15–19].

By combining the colorimetric detection modalities, paper-based colorimetric POC diagnostics offer distinct advantages such as affordability, portability, and visual readout, enabling easy interpretation of results without the need for additional pieces of equipment [20–22]. Despite their significant advantages, colorimetric assays on paper do have certain limitations. One of the primary challenges is their inherent limitation in sensitivity, particularly when detecting biomarkers at low concentrations [23]. To overcome these limitations and to enhance the sensitivity of colorimetric assays on paper, researchers have pioneered sophisticated techniques that incorporate signal amplification strategies driven by rapid advancements in novel materials and nanotechnology [24]. A significant focus has been placed on enhancing the signal output through a meticulous control of the physicochemical properties of nanoparticles, such as their size and shape and functionalization of nanocomposites with polymers and the utilization of enzyme-mimicking noble metal nanoparticles as an effective means to amplify the colorimetric signal [25].

Furthermore, in order to enhance colorimetric signals on paper, other techniques such as liquid evaporation and electrokinetic methods have been utilized. Electrokinetic approaches majorly comprise field-amplified sample stacking (FASS), ion concentration polarization (ICP), and isotachophoresis (ITP) [26]. However, these aforementioned techniques are complex and time-consuming and require specialized equipment and expertise[27–29]. Furthermore, the need for reagents and additional steps for electrode preparation (in the case of electrokinetic methods) and maintenance adds further complexity and cost [30, 31]. These limitations highlight the urgent need to devise simplified and resilient strategies for sensitivity enhancement in colorimetric paper-based diagnostics[32, 33].

**Fig. 1:**
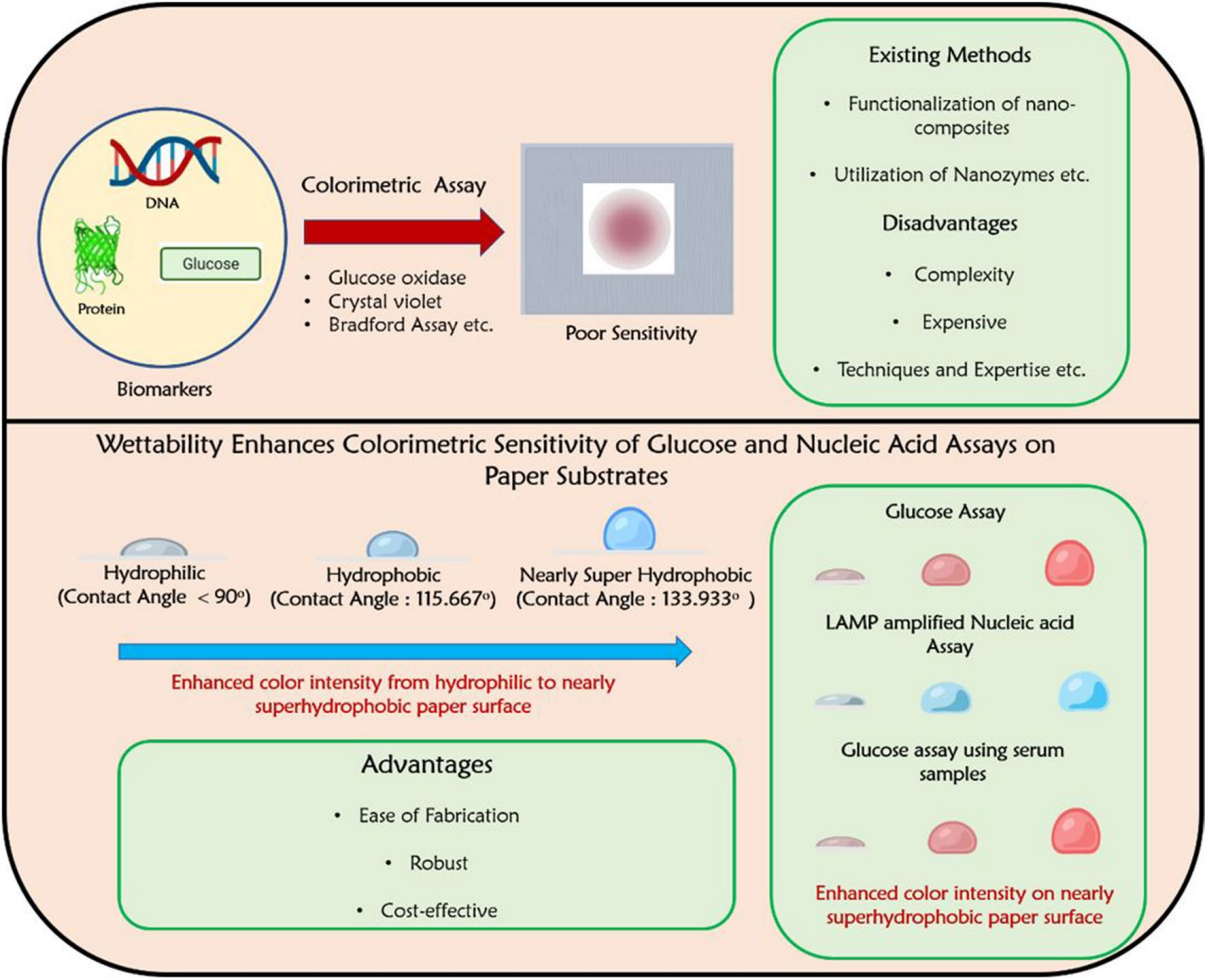
Schematic illustration showing the wettability tuning of paper-spot devices for enhanced colorimetric sensitivity of glucose and nucleic acid assays

In this manuscript, for the first time, we present an innovative approach to enhance the sensitivity of colorimetric assays, specifically for the detection of glucose and *Candida albicans* DNA. We achieve this by precisely tuning the wettability of paper-based devices. Modifying the surface properties of the paper substrate allows us to achieve hydrophobic and nearly superhydrophobic characteristics, resulting in remarkable improvements in assay performance. To create hydrophobic surfaces, we employ candle scratching and heating techniques thereby carefully controlling the surface roughness. Additionally, we utilized commercially available spray onto the candle-scratched paper to create nearly superhydrophobic surfaces. Through glucose oxidase and colorimetric nucleic acid assays conducted on the modified surfaces (tuning wettability), we observed a significant enhancement in colorimetric sensitivity (Fig. 1). Furthermore, we assess the clinical applicability of this approach using serum samples from patients with varying glucose levels. The simplicity of our method allows for easy implementation, even by non-experts, and the visual readout enables rapid interpretation of results without the need for expensive equipment. Besides, the approach enables the detection of target biomolecules even at low concentrations, which is of paramount importance for early disease diagnosis and monitoring. Besides, the modified paper surfaces also exhibit enhanced stability and robustness, ensuring reliable assays under varying environmental conditions [34–37]. Furthermore, the improved sensitivity reduces the required sample volume for accurate detection, which is beneficial for handling limited or precious samples. By addressing these limitations, our study aims to bridge the gap between sophisticated, expensive diagnostic methods and the urgent need for accessible and affordable diagnostics, particularly in resource-limited settings.

## Materials and methods

### Materials

Grade 1 Whatman (R) filter paper (thickness 180 µm and pore size, 11 µm) was obtained from GE Life Sciences. Scotch BOPP transparent (50.8 mm × 50 meter) tape manufactured by 3 M™, India, was procured from a local stationary shop. Candle sticks were purchased from a local vendor. Neverwet superhydrophobic spray manufactured by Rust-Oleum was purchased from Amazon, India. Glucose (S.L) reagent was purchased from Agappe Diagnostics Ltd. Crystal violet and nuclease-free water were purchased from Himedia. Sodium sulfite (Na_2_SO_3_) was purchased from SRL, India. The *Candida albicans* (ATCC 24433) culture was collected from the Department of Microbiology, Kasturba Medical College, Manipal. Anonymized clinical serum samples (total of 12 samples comprising triplicates of each concentration, specifically, 60 mg/dL, 90 mg/dL, 120 mg/dL, and 150 mg/dL) validated using EM 360 autoanalyzer were collected from the Department of Biochemistry, Kasturba Medical College, Manipal.

## Methods

### Device fabrication

Whatman® filter paper (grade 1) was used in this study. Paper-spot devices were created by punching the paper using a 4 mm diameter single-hole punching machine. In order to introduce a hydrophobic coating, the paper devices were gently scratched with a candle and subsequently placed inside a hot air oven set at 100 °C for 15 min. This step ensured the penetration of paraffin wax into the perforated pores of the paper. Consequently, these devices were affixed onto a scotch transparent tape. To impart a nearly superhydrophobic coating to the paper devices, a fresh set of 4 mm diameter holes were punched, and the paper spots were gently scratched with a candle as before and incubated at 100 °C for 15 min. After the incubation, these devices were taped. To further enhance the water-repellent properties, and achieve a nearly superhydrophobic surface, 3 μL of Neverwet superhydrophobic spray was sprayed onto these paper devices. The devices were then allowed to air dry at room temperature for 30 min.

### Contact angle measurement

To evaluate the hydrophilic, hydrophobic, and nearly superhydrophobic properties of the paper devices, a contact angle measurement was performed. A water droplet of 10 µL was carefully placed on each surface, and horizontal images of the water droplets formed on the paper devices were captured using a DSLR camera (Canon EOS 80D). The contact angle was quantitatively analyzed using Fiji (ImageJ) software. In the analysis, five reference points were manually selected along the periphery of the droplets, and an ellipse was created using the manual point procedure. Furthermore, the contact angle was determined using the following equation:

$$\mathrm{Contact\; angle }\left(\mathrm{CA}\right)=180-\mathrm{\theta E}$$

where $\uptheta$ E represents the angle measured from the created ellipse. All the trials were done in triplicate. The mean contact angle and the standard deviation (SD) for hydrophilic, hydrophobic, and nearly superhydrophobic paper surfaces were calculated and tabulated.

### Glucose oxidase assay with standard glucose samples

To evaluate the colorimetric sensitivity enhancement of the glucose assay on the fabricated paper spots, we performed the glucose oxidase assay following the established protocol by Agappe, India [38]. Initially, we prepared a series of standard glucose concentrations, specifically 60 mg/dL, 80 mg/dL, 100 mg/dL, 120 mg/dL, and 150 mg/dL. In amber-colored vials, we added 1000 µL of glucose (S.L) reagent and subsequently added 10 µL of each standard glucose concentration to the respective vials. The vials were then incubated at 37 °C for 10 min in a dry bath. After the incubation period, 10 µL of the reaction product was added to each hydrophilic, hydrophobic, and nearly superhydrophobic paper-spot device. To ensure consistent and uniform image capturing of the fabricated paper-spot devices, a Canon EOS 80D DSLR camera set to AV mode was used. The picture style was set in “daylight” to portray the true colors of the colorimetric reaction products, avoiding any artificial color shifts or biases that could influence our analysis. The ISO rate was fixed at 600 to maintain uniform sensitivity to light and consistent exposure levels across all captured images. To achieve the desired focus on the paper-spot devices and control the depth of field, the aperture was set to f/3.5. Additionally, the color saturation and contrast settings were left untouched to preserve the vividness and dynamic range of the images. The images of the colorimetric test samples, including the controls, were inverted, and processed using the ImageJ software. The inverted images were split into red (R), green (G), and blue (B) channels, and a line zone was selected in the blue (B) channel image to quantify the colorimetric response, specifically in the region where the colorimetric reaction product is concentrated [39, 40]. The intensity measurements were performed in triplicate for each glucose concentration.

### Loop-mediated isothermal amplification (LAMP)

The conserved sequence of *Candida albicans*, the Internal Transcribed Spacer-2 (ITS-2) sequence, was amplified by loop-mediated isothermal amplification (LAMP). The primer sequence for *Candida albicans* ITS-2 was obtained from previous studies (Supplementary Table 1). All the samples were prepared in triplicates. The LAMP reaction was carried out in a final volume of 50 μL comprising five different primers comprising of forward inner primer (FIP) (2 μM), backward inner primer (BIP) (2 μM), loop backward (LB) (0.33 μM), forward (F3) (0.167 μM) and backward (B3) (0.167 μM) (Proteogen Biosciences), 1.4 mM dNTPs (New England Biolabs, USA), 1 × isothermal amplification buffer (New England Biolabs, USA), 6 mM MgSO_4_ (New England Biolabs, USA), 8 U of Bst 2.0 Warm Start DNA polymerase (New England Biolabs, USA), and 2 μL of DNA template. The reaction was run at 65 °C for 60 min and was terminated at 80 °C for 5 min in a thermocycler.

### Nucleic acid assay

Firstly, for the nucleic acid assay, ITS-2 amplicon sequences of different concentrations, specifically, 80 ng/µL, 100 ng/µL, 200 ng/µL, and 500 ng/µL from *Candida albicans*, were prepared using nuclease-free water and stored at 4^ο^C. Crystal violet (CV) solution was prepared using 0.5 mM CV and 50 mM Tris–Hcl (pH 8.8). From these solutions, 40 µL of CV and 10 µL of 30 mM sodium sulfite were mixed to yield a leucocrystal violet (LCV) solution. Next, the LCV control solution was prepared by mixing 50 μL of LCV solution with an equivalent volume of distilled water and reacting at room temperature for 10 min. The LCV + DNA solution was prepared using the same protocol as that for the LCV solution, with the only difference being the utilization of previously prepared amplicon samples at different concentrations instead of using distilled water. Ten microliters of these reaction products was added to different paper-spot devices having hydrophilic, hydrophobic, and nearly superhydrophobic surfaces. For consistent and uniform image capturing of the paper-spot devices, the same imaging parameters (as described previously) were set in the camera. After capturing the images of the colorimetric test samples, including the controls, image inversion was performed and processed using the ImageJ software. These inverted images were split into red (R), green (G), and blue (B) channels, and selected a line zone in the green (G) channel image to quantify the colorimetric response, specifically in the region where the colorimetric reaction product is concentrated [39, 40]. To ensure accuracy, the intensity measurements were performed in triplicate for each nucleic acid concentration.

### Evaluation of serum glucose concentration on varying hydrophobic paper spots

To assess the clinical applicability of our developed device, a glucose oxidase assay was conducted (as described before) by collecting serum samples from patients with varying glucose levels and analyzing them using our devices having different hydrophobicities. The assay was carried out on anonymized clinical serum samples from patients having different concentrations of fasting blood glucose (FBG), specifically, 60 mg/dL, 90 mg/dL, 120 mg/dL, and 150 mg/dL. To achieve consistent and uniform image capturing of the fabricated paper-spot devices, similar imaging parameters as previously described were set in the camera. After capturing the images of the colorimetric test samples, including the controls, we performed image inversion and processed them using the ImageJ software. Subsequently, the inverted images of the clinical serum samples were split into red (R), green (G), and blue (B) channels and selected a line zone in the blue (B) channel image to quantify the colorimetric response, particularly in the region where the colorimetric reaction product is concentrated [39, 40]. To ensure accuracy, intensity measurements were performed in triplicate for each serum concentration.

### Statistical analysis

Experimental data obtained from standard glucose; nucleic acids and serum glucose assays were represented as mean ± standard deviation (SD). The statistical significance of B channel intensities exhibited by glucose droplets (both standard and serum glucose) and G channel intensities of the nucleic acid samples on all hydrophilic, hydrophobic, and nearly superhydrophobic paper-spot devices were determined using the one-way ANOVA test with Tukey’s post hoc analysis in GraphPad Prism 8 software. A significance level of *p* < 0.05 was considered statistically significant.

### Calculating limit of detection (LOD)

The limit of detection was calculated by using the below-given equation.

$$LOD=\frac{3.3\; \mathbf{X}\;\mathbf{S}\mathbf{D}}{\sigma }$$

where SD is the standard deviation;* σ* is the slope.

## Results and discussion

### Water contact angle measurement of hydrophobic and nearly superhydrophobic surfaces

In our study, we aimed to investigate the behavior of glucose and nucleic acid assays on different paper surfaces, including hydrophilic, hydrophobic, and nearly superhydrophobic surfaces. The water contact angle (WCA) formed by water droplets on each of these paper surfaces was measured using ImageJ software. No contact angle was formed for the hydrophilic paper surface, whereas the hydrophobic paper surface and nearly superhydrophobic paper surface have shown the contact angles 115.667° and 133.933° respectively (Table 1). The observed contact angles align with the theoretical understanding of contact angle formation, which is based on the intermolecular forces between the water droplet and the paper surfaces [41]. The contact angle, also known as a wetting angle, is the angle formed at the point, where a liquid drop contacts a solid surface [42]. This phenomenon is influenced by various factors, including the surface tension of the liquid, the surface energy of the solid, and the topography of the surface [43].

**Table 1:** The measured mean contact angle and standard deviation (SD) values for hydrophilic, hydrophobic, and nearly superhydrophobic paper surfaces

Sl. no	Type of paper surface	Mean contact angle	SD
1.	Hydrophilic	No contact angle was observed	-
2.	Hydrophobic	115.667^ο^	5.23
3.	Nearly superhydrophobic	133.933^ο^	3.72

**Fig. 2:**
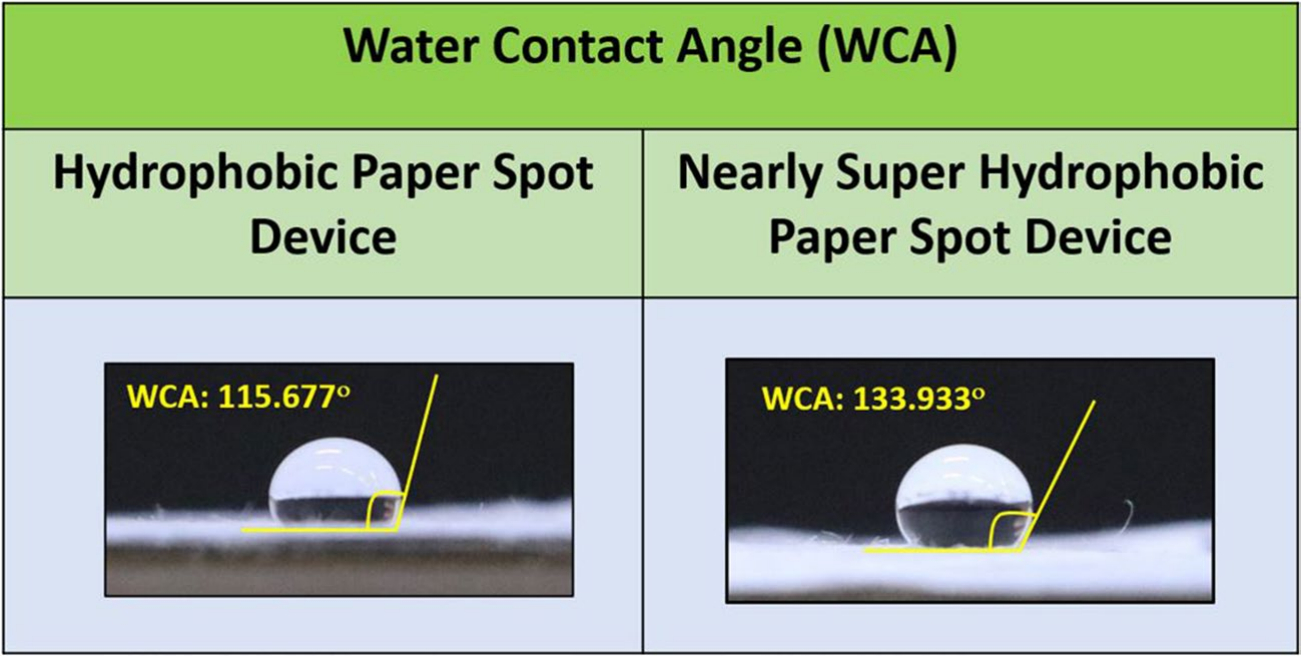
Water contact angle (WCA) measured for hydrophobic and nearly superhydrophobic paper surfaces

The hydrophilic paper surface exhibited a smaller contact angle, indicating a high affinity between the water droplet and the surface. This is in line with the concept that hydrophilic surfaces have a higher surface energy than the surface tension of the liquid [44]. Consequently, the water droplet spreads out and wets the surface, leading to a relatively smaller contact angle. On the other hand, the hydrophobic paper surface showed a contact angle of 115.667° (Fig. 2). This larger contact angle compared to the hydrophilic paper surface suggests that the surface energy of the paper is lower than the surface tension of the water droplet [45]. As a result, the droplet tends to bead up and form a more spherical shape, minimizing contact with the hydrophobic surface. Nearly superhydrophobic paper surface exhibited the largest contact angle of 133.933° (Fig. 2). This is attributed to the surface possessing relatively low surface energy compared to the surface tension of the water and intricate surface topography [46]. These parameters together contribute to a non-wetting behavior. Consequently, the water droplet sits on top of the surface structure, minimizing the contact area and resulting in a larger contact angle.

#### Colorimetric standard glucose assay on hydrophilic, hydrophobic, and nearly superhydrophobic surfaces

Glucose, a vital energy source for the body, is crucial in the diagnosis and management of diabetes mellitus. Plasma and serum glucose levels are commonly assessed using a colorimetric assay that involves the conversion of glucose and oxygen to gluconate and hydrogen peroxide (H_2_O_2_) by glucose oxidase. The presence of H_2_O_2_ leads to the formation of a colored complex, quinonimine, through a reaction with 4-amino antipyrine and a phenolic compound [38]. Typically, this colored complex is measured spectrophotometrically within the wavelength range of 490–550 nm. In our study, we sought to enhance the colorimetric sensitivity of the glucose oxidase assay by adjusting the wettability of paper surfaces using different concentrations of standard glucose. Our findings revealed that among the selected concentrations of glucose, the blue channel intensity was observed in the order hydrophilic < hydrophobic < nearly superhydrophobic paper surfaces.

**Fig. 3:**
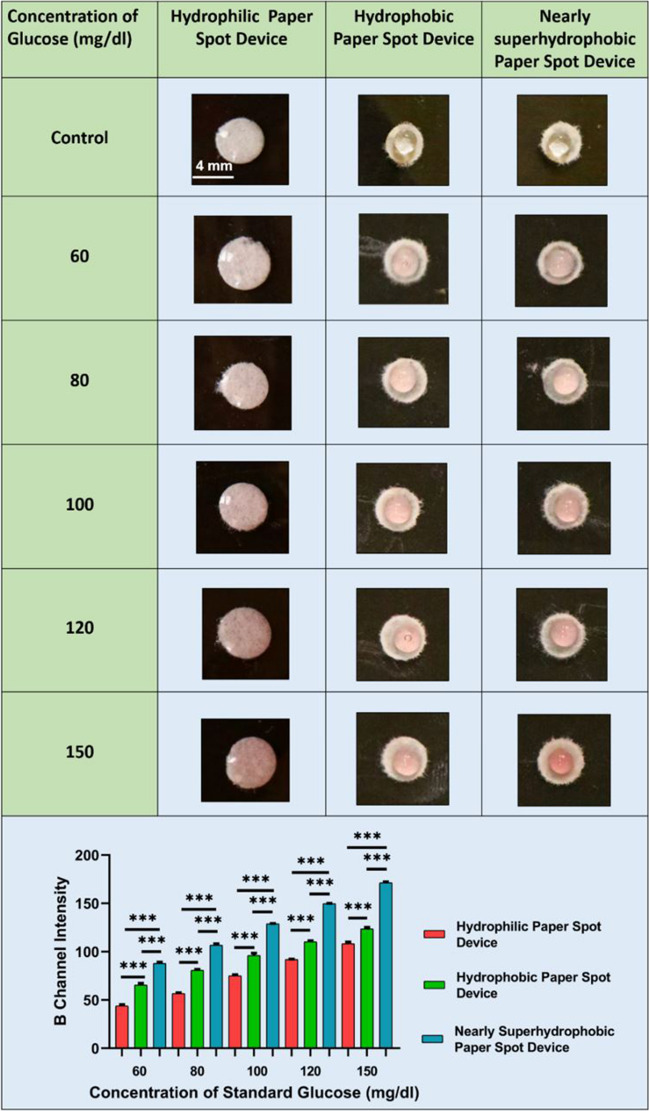
Glucose oxidase assay performed for different standard glucose concentrations on different paper surfaces and their respective blue (B) channel intensities. Level of significance *p < 0.05, **p < 0.01, and ***p < 0.001 for comparison between the samples

The hydrophilic paper surface displayed the lowest blue channel intensity, indicating a lower colorimetric sensitivity. This difference can be attributed to the high wettability of the hydrophilic surface, causing the reaction product to spread and disperse over a larger area [35], resulting in a lower intensity. Furthermore, the hydrophobic paper surface exhibited a relatively higher blue channel intensity compared to the hydrophilic paper devices, indicating a moderate enhancement in colorimetric sensitivity (Fig. 3, top). This enhancement is corroborated by the molecular crowding effect, which restricts the spreading and diffusion of the glucose reaction product to a controlled extent [47]. As a result, the reaction product remains more confined within the paper surface, leading to a noticeable increase in the intensity. We also hypothesize that pore blockage on the paper surface (by hydrophobic agents) restricts the axial movement of the reaction product, allowing it to be more concentrated within the hydrophobic paper surface. The accumulation of the analyte at the sample loading zone contributes to a stronger colorimetric signal, thereby enhancing the color intensity and sensitivity [48]. The nearly superhydrophobic paper surfaces exhibited the highest colorimetric sensitivity, which can be attributed to several factors [49]. Firstly, a very low surface energy of the nearly superhydrophobic surface combined with its intricate surface topography creates a non-wetting behavior. This behavior confines the reaction product to remain localized in a smaller area upon addition, resulting in higher intensity measurements.

Generally, distinguishing narrow concentrations of glucose solely based on intensity is challenging in paper-based colorimetric assays [50, 51]. This limitation leads to false negative or false positive results. However, by incorporating superhydrophobic materials, it becomes more viable to differentiate close glucose concentrations solely based on color intensity. Additionally, the statistical analysis conducted on the blue channel intensities of different concentrations of glucose droplets added to hydrophilic, hydrophobic, and nearly superhydrophobic paper surfaces revealed significant differences in intensity (Fig. 3, bottom). Specifically, the intensity of droplets on both hydrophobic and nearly superhydrophobic paper surfaces was significantly higher compared to the hydrophilic surface across all glucose concentrations.

#### Colorimetric nucleic acid assay on hydrophilic, hydrophobic, and nearly superhydrophobic surfaces

Furthermore, to assess the potential application of our approach in colorimetric nucleic acid assays, we conducted experiments using ITS-2 amplicons isolated from *Candida albicans* [52]. In this assay, crystal violet (CV) was utilized as an indicator to detect the presence of dsDNA. Crystal violet, a triphenylmethane dye with a p-quinoid group acting as a chromophore, has a pronounced affinity for double-stranded DNA (dsDNA). Initially, when sodium sulfite (Na_2_SO_3_) is added to crystal violet, CV undergoes a conversion into leucocrystal violet (LCV), which is colorless [53]. Upon introducing dsDNA, the positively charged quinoid of CV interacts with the dsDNA through electrostatic attraction which results in a violet color [54]. This colorimetric response serves as a reliable and sensitive method for detecting the presence of dsDNA.

**Fig. 4:**
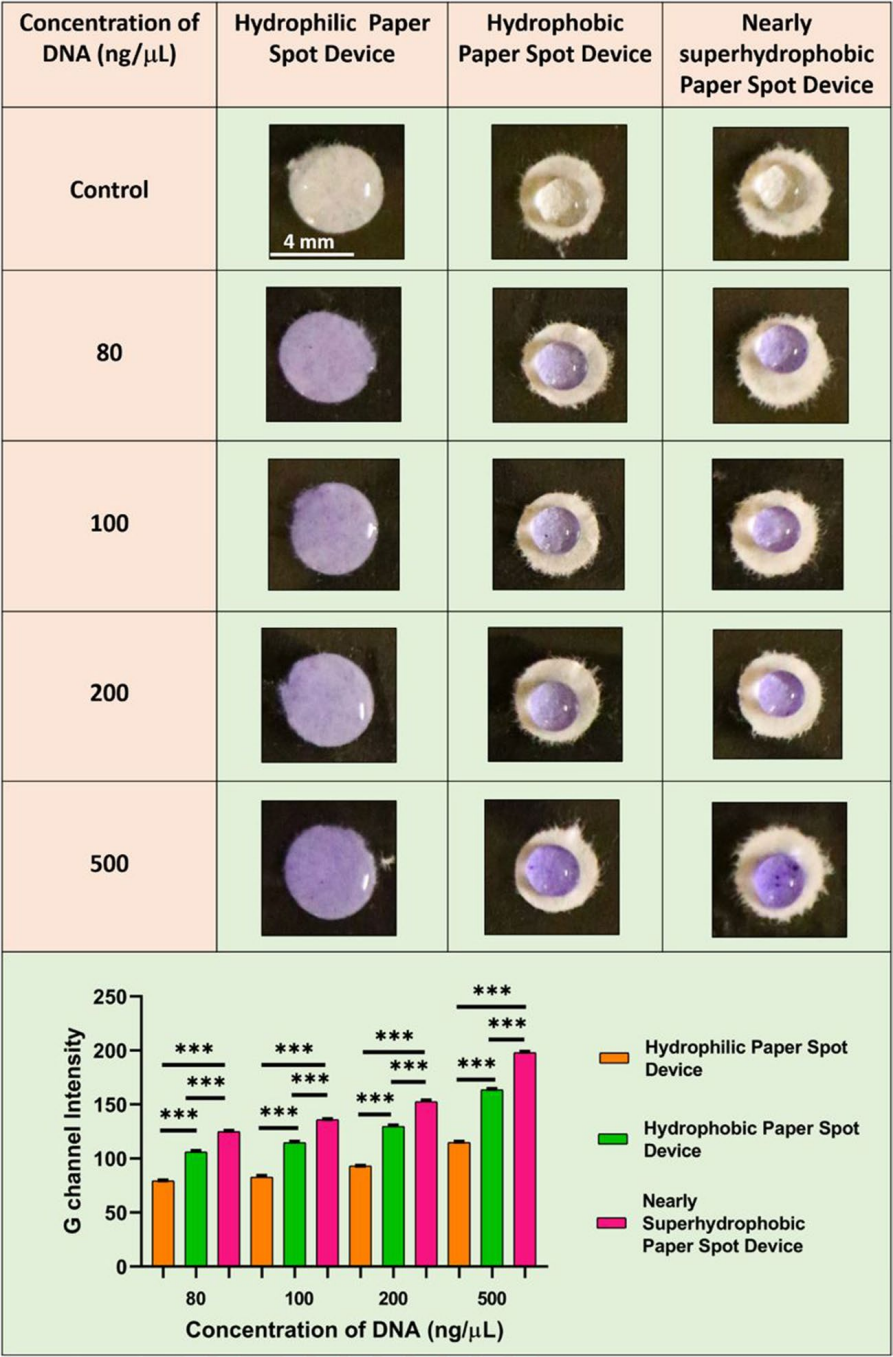
Nucleic acid assay for different concentrations of dsDNA on different paper surfaces and their respective green (G) channel intensities. Level of significance *p < 0.05, **p < 0.01, and ***p < 0.001 for comparison between the samples

Hydrophilic surfaces characterized by high wettability cause the LCV-DNA mixture to spread extensively, leading to a larger area of dispersion and consequently a lower color intensity (Fig. 4, top). Whereas, hydrophobic surfaces exhibit a moderate intensity compared to hydrophilic surfaces. This enhancement in intensity is corroborated by the previously described molecular crowding effect, which restricts the spreading and diffusion of the LCV-DNA mixture [47]. As a result, the reaction product remains more confined within the paper surface, leading to a noticeable increase in intensity. Similarly, the nearly superhydrophobic surfaces exhibit the most pronounced enhancement in colorimetric response and improved detection sensitivity. This is primarily due to the higher contact angle and reduced spreading of the LCV-DNA mixture on the superhydrophobic surface. The low surface energy and intricate surface topography of the superhydrophobic surface create a non-wetting behavior, allowing the reaction product to remain localized in a smaller area [55]. These observations were further validated through statistical analysis of green (G) channel intensities of droplets added to these three distinct surfaces (Fig. 4, bottom).

**Fig. 5:**
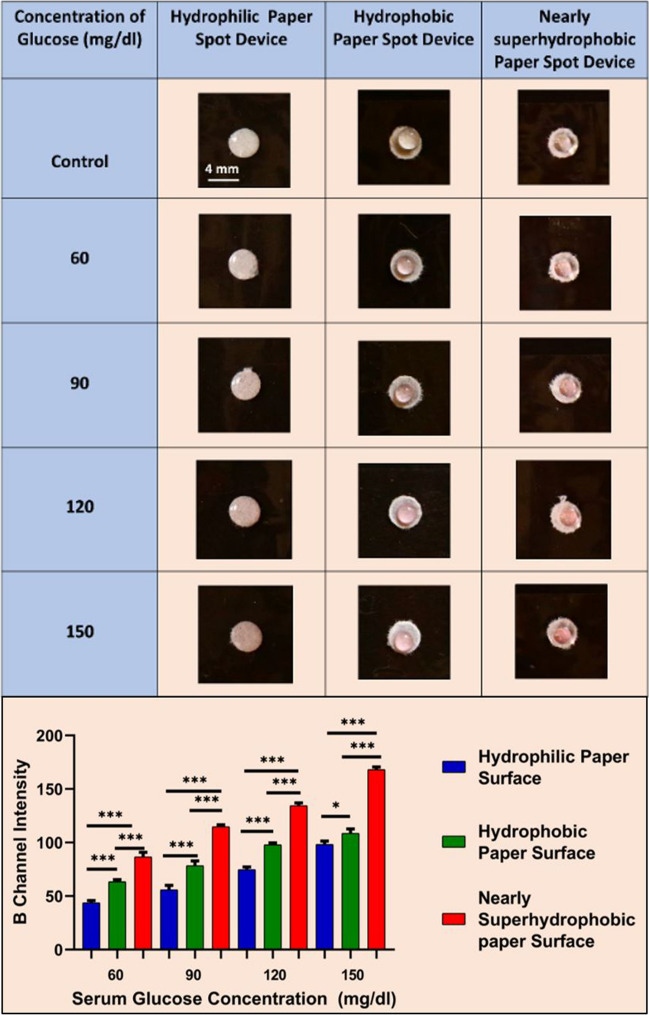
Glucose oxidase assay for different concentrations of glucose in serum samples on different paper surfaces and their respective blue (B) channel intensities. Level of significance *p < 0.05, **p < 0.01, and ***p < 0.001 for comparison between the samples

Furthermore, the practicality of this approach was validated using anonymized clinical serum samples (undergone prior validation using EM 360 autoanalyzer) of normal and diabetic patients. It was observed that the nearly superhydrophobic paper surfaces outperformed hydrophobic and hydrophilic paper surfaces (Fig. 5, top). More importantly, the nearly superhydrophobic surface yielded better distinguishable color intensity for narrow glucose concentrations. This was supported through statistical analysis of respective blue (B) channel intensities exhibited by serum samples on hydrophilic, hydrophobic, and nearly superhydrophobic paper surfaces (Fig. 5, bottom).

#### Limit of detection (LOD)

The limit of detection (LOD) for nucleic acid, standard glucose, and clinical serum samples was determined from the standard calibration plot. The change in the B and G channel intensities was plotted against respective concentrations of the analytes, ranging from 80 to 500 ng/µL for nucleic acid and 60 to 150 mg/dL for both standard glucose and serum glucose samples. Supplementary Fig. 1 illustrates a linear relationship between the concentration of analytes and their respective intensities. The LOD of nucleic acid on the nearly superhydrophobic surfaces (16.15 ng/µL) was found to be almost four-fold lower than that on the hydrophilic surfaces (60.08 ng/µL). Similarly, the LODs of standard glucose and serum glucose samples revealed a two-fold lower value on the nearly superhydrophobic surfaces compared to the hydrophilic surfaces. The calculated LODs of all the analytes on various surfaces are summarized in Table 2.

**Table 2:** Measured LOD for nucleic acid, standard glucose, and serum glucose on different paper surfaces

Analytes	Surface	LOD	*R*^2^
Nucleic acid	Hydrophilic	60.08 ng/μL	0.969
	Hydrophobic	36.52 ng/μL	0.982
	Nearly superhydrophobic	16.15 ng/μL	0.989
Standard glucose	Hydrophilic	12.56 mg/dL	0.985
	Hydrophobic	10.03 mg/dL	0.992
	Nearly superhydrophobic	6.19 mg/dL	0.989
Serum glucose	Hydrophilic	14.37 mg/dL	0.976
	Hydrophobic	9.23 mg/dL	0.986
	Nearly superhydrophobic	7.52 mg/dL	0.982

## Conclusion

For the first time, we have demonstrated that harnessing the wettability of the paper surfaces in colorimetric assays holds great promise for improving the intensity. For nearly superhydrophobic surfaces, we have achieved a two-fold increase in the sensitivity of standard and serum glucose concentrations. Similarly, we have accomplished a four-fold increase in the sensitivity of LAMP amplicons of the fungus, *Candida albicans*. The superior performance of the nearly superhydrophobic paper surfaces in differentiating narrow analyte concentrations is due to its water-repelling nature, which promotes localized concentration of the assay product which in turn leads to the accumulation of reaction product with better colorimetric signal. Furthermore, the relatively lower surface energy compared to the surface tension of the reaction products and intricate surface topology of nearly superhydrophobic surfaces resulted in amplified colorimetric signals. This approach presents a practical and reliable solution for distinguishing analyte concentrations using simulated as well as clinical samples. Exploring the compatibility and applicability of superhydrophobic surfaces in diverse diagnostic scenarios can lead to the development of innovative and reliable diagnostic devices with improved performance and accuracy.

### Supplementary Information

Below is the link to the electronic supplementary material.

Supplementary file1 (DOCX 264 KB)

## Declarations

### Source of biological material

Anonymized serum samples were collected from the Department of Biochemistry, Kasturba Medical College, Manipal 576104.
