# Supplementary material for: Tuning Hydrophobicity of Paper Substrates for Effective Colorimetric detection of Glucose and Nucleic acids
Source: Anal Bioanal Chem. 2023 Sep 4;415(26):6449–60. doi: 10.1007/s00216-023-04921-2 (PMC10567893; doi:10.1007/s00216-023-04921-2)
Supplement: Supplementary file 1 — Supplementary file1 (DOCX 264 KB) [file 216_2023_4921_MOESM1_ESM.docx]

**Supporting Information**

**Tuning Hydrophobicity of Paper Substrates for Effective Colorimetric Detection of Glucose and Nucleic Acids**

Sujesh Sudarsan^a±^, Prashil Shetty ^a±^, Raja Chinnappan^b^ and Naresh Kumar Mani^a*^

^a^Microfluidics, Sensors and Diagnostics (µSenD) Laboratory, Centre for Microfluidics, Biomarkers, Photoceutics and Sensors (µBioPS), Department of Biotechnology, Manipal Institute of Technology, Manipal Academy of Higher Education, Manipal, Karnataka 576104, India.

^b^ College of Medicine, Alfaisal University, Riyadh 11533, Saudi Arabia

**Corresponding author:** Email: <naresh.mani@manipal.edu>, [maninaresh@gmail.com](mailto:maninaresh@gmail.com)

***Supplementary Table 1:*** *Primer sequence of FIP, BIP, LB, B3 and F3 that recognize specific target gene, ITS-2 of Candida albicans*

| Primers | Sequence | Length (bp) |
| --- | --- | --- |
| FIP | CTACCGTCTTTCAAGCAAACCCATGAGCGTCGTTTCTCCCT | 41 |
| BIP | TTGACAATGGCTTAGGTCTAACCAAAAGATATACGTGGTGGACGTTAC | 48 |
| LB | CTCAACACCAAACCCAGCGG | 20 |
| F3 | TCTGGTATTCCGGAGGGC | 18 |
| B3 | AGTCCTACCTGATTTGAGGT | 20 |


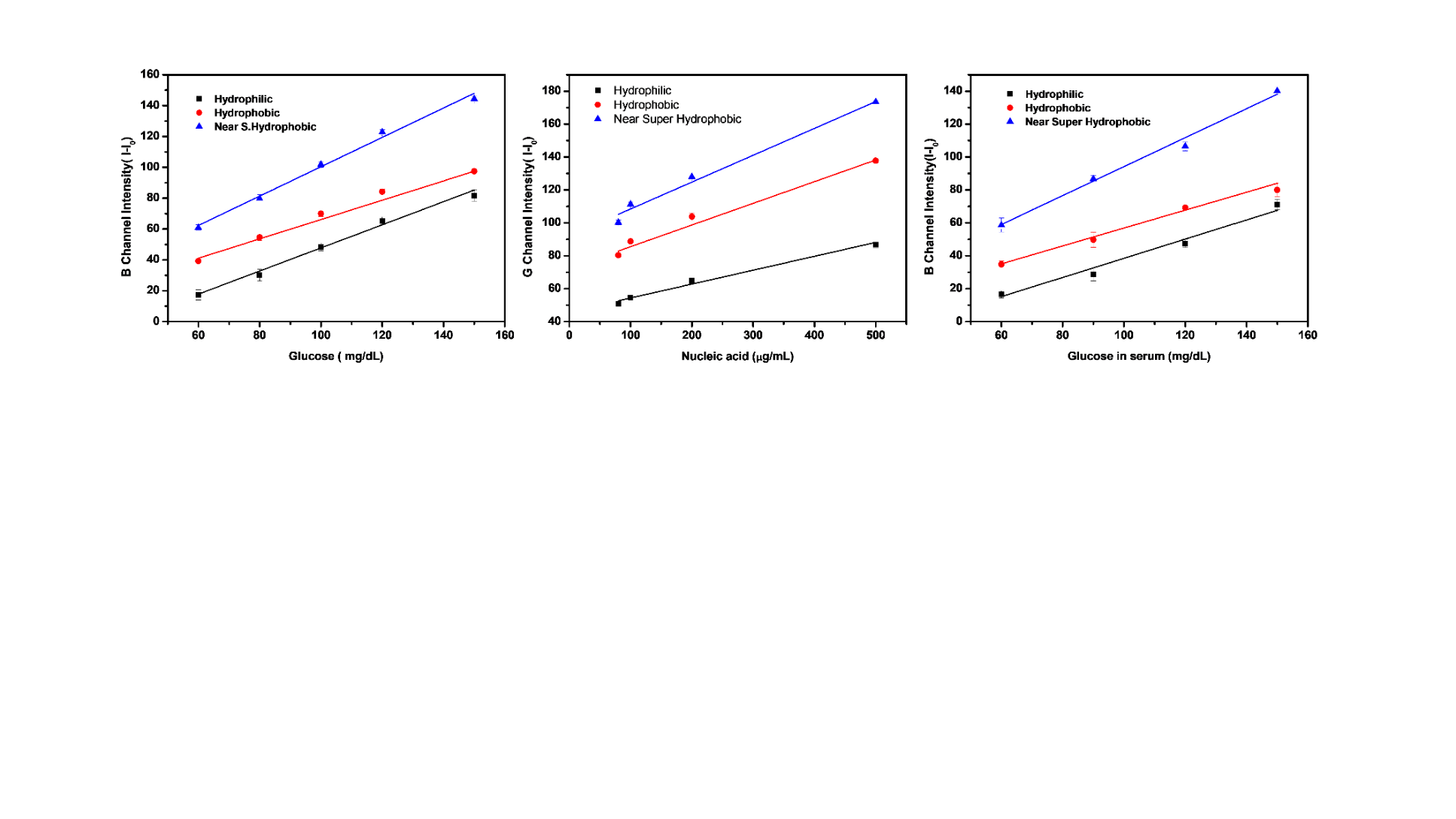


***Supplementary Figure 1:*** *LODs for standard glucose, nucleic Acid and serum glucose*
